# Acceptance of euthanasia by students of selected study disciplines at universities in Lublin, Poland

**DOI:** 10.1186/s12910-024-01071-7

**Published:** 2024-07-26

**Authors:** Stanisław Lachowski, Bogusława Lachowska, Magdalena Florek-Łuszczki

**Affiliations:** 1 University of Maria Curie-Skłodowska, Lublin, Poland; 2 The John Paul II Catholic University of Lublin, Lublin, Poland; 3 Institute of Rural Health, Lublin, Poland

**Keywords:** Euthanasia, Students, Study disciplines

## Abstract

**Background:**

In the context of discussions between supporters and opponents of euthanasia, and legal regulations regarding this type of practices, the attitude of young people with respect to this phenomenon is a very interesting issue. According to Polish law, euthanasia is prohibited. The aim of this study was to determine the degree of acceptance of euthanasia among students from Polish universities across three different fields of study: psychology, medicine, and economic-technical disciplines, and to identify the factors associated with the acceptance of this phenomenon.

**Methods:**

The study included 627 persons studying in Lublin, Poland: medicine (280), psychology (170), and economic-technical studies (177). The study was conducted as a survey using questionnaire containing items concerning students’ attitudes towards euthanasia. The analysis of the collected data was conducted using the SPSS software (version 29) with the following methods: Chi^2^, Student’s t-test, Phi test, Cramer’s V test, Kolmogorov-Smirnov test, one-way ANOVA with Dunnett’s post hoc test, linear regression analysis, and categorical regression analysis using optimal scaling (CATREG).

**Results:**

Nearly half of the students participating in the study (45.9%) rated the practice of euthanasia as decidedly negative. The highest number of strongly negative evaluations was found among psychology students, and the least among students of economic-technical disciplines. The level of acceptance of euthanasia is significantly associated with religious involvement and studying psychology. Being religious and being a psychology student both contribute to lower acceptance of euthanasia and a lower willingness to consent to euthanasia. Consent to euthanasia is more commonly declared by individuals with experience of living with elderly people.

**Conclusions:**

Although nearly half of the respondents expressed a negative attitude towards euthanasia, considering the secularization process among Polish youth, it can be assumed that the level of acceptance of euthanasia in this social group will increase. The lower level of acceptance of euthanasia among psychology and medical students compared to students of economic-technical disciplines suggests that the curricula of these studies present alternative solutions to the problems of terminally ill patients other than euthanasia.

**Supplementary Information:**

The online version contains supplementary material available at 10.1186/s12910-024-01071-7.

## Background

Euthanasia and other related phenomena are increasingly becoming subjects of bioethical discussions [1, 2]. One of the first authors expressing approval for the active assistance of the caregiver/physician during the killing of a patient was Samuel Williams in the USA. In 1870, Williams for the first time proposed the use of anaesthetics and morphine to terminate the life of patients suffering from incurable illnesses [3]. The assistance of a physician or caregiver were supposed to consist in the administration, at the patient’s request, of an agent which will cause a quick and painless death. Simultaneously, according to Williams, such an action should not be considered as a manifestation of mercy towards the suffering person, but as a rational choice, or even a duty of the caregiver. This way of understanding euthanasia, referred to as the eugenic trend, was developed by the representatives of Nazi Germany who, based on these views led to extermination of, in their opinion, inconvenient, unnecessary groups of citizens, later extended to include specific national groups.

Nowadays, there are many definitions of the term euthanasia. The American Medical Association’s Council on Ethical and Judicial Affairs, while seeking the most common definition, stated that euthanasia is the act of intentionally causing death of a hopelessly ill and suffering person in a quick and painless way, guided by the good of the person [4]. This definition contains two important characteristics of euthanasia: (1) consciously taking the life of another human being; and (2) the reason for ending another person’s life is his/her well-being.

In the context of ongoing discussions on euthanasia, Radbruch, Leget, Bahr, Müller-Busch, Ellershaw, de Conno, and Vanden Berghe on behalf of the board members of the European Association for Palliative Care [5] define euthanasia and other terms that appear in the literature and public debate related to euthanasia. They highlight the differences and similarities between them. They emphasize that euthanasia physician assisted suicide, withholding/withdrawing of treatment, palliative sedation are different concepts. These phenomena are frequently the subject of research [6, 7]. Radbruch, Leget, Bahr, Müller-Busch, Ellershaw, de Conno and Vanden Berghe defines euthanasia as a situation in which „a physician (or other person) intentionally killing a person by the administration of drugs, at that person’s voluntary and competent request” [5, p. 108]. From this definition, it follows that euthanasia is always active, and the term “passive euthanasia” is a contradiction in itself. Moreover, euthanasia can be voluntary only [8]. This definition raises controversy for some, for instance, due to the use of the word “killing” and the belief that only a physician should be authorized to perform euthanasia. Assisted suicide is defined as a situation in which: “a person intentionally helping another person to terminate his or her life, at that person’s voluntary and competent request” [5, p. 108]. In this case, the authority of action (unlike in euthanasia) and the decision-making process (similar to euthanasia) remain with the person who wants to end their life. Physician-assisted suicide (PAS), on the other hand, refers to a situation where a physician intentionally helps a person terminate their life by providing drugs for self-administration, at that person’s voluntary and competent request [5, p. 108]. The medicalization of PAS raises controversy, partly because it is seen as part of the transformation of medicine from a caring profession into a business aimed at meeting the demand for medical services [9]. On the other hand, there are reports of alternative concepts where euthanasia and assisted suicide are performed by non-physicians [10]. Another phenomenon defined by the authors is the withholding or withdrawing of treatment from a person due to the futility of the treatment or at that person’s voluntary and competent request (TD) [5, p. 108]. NTD, unlike euthanasia, does not aim to hasten death but rather allows for imminent death to occur naturally. It is considered an acceptance of death as a natural phenomenon, involving the omission of futile, burdensome, or unwanted life-prolonging procedures [11]. A completely different phenomenon is palliative sedation, defined as “the monitored use of medications intended to induce a state of decreased or absent awareness (unconsciousness) in order to alleviate intractable suffering in a manner that is ethically acceptable to the patient, family, and healthcare providers.” [12, p. 109]. Applied in appropriate situations, it is an accepted, ethical practice. It is believed that palliative care, when provided until the end of life, is never futile by definition and may be an option for many patients in states where they may request euthanasia or PAS [12].

The legal regulations concerning the application of euthanasia are highly diverse. In some countries, such as the Netherlands, Belgium, Luxembourg, Switzerland, and Spain, euthanasia is legalized [13, 14]. In these countries, physician-assisted suicide is also legal. Physician-assisted suicide, excluding euthanasia, is legal in places like five states in the USA (Oregon, Washington, Montana, Vermont, and California), Colombia, and Canada [15], as well as in Germany and Italy [16], New Zealand [17]. In countries where euthanasia has been legalized, the number of such procedures is systematically increasing. An example of the high dynamism of this phenomenon is Belgium. One year after the legalization of euthanasia, four procedures were performed, and by 2023, the number had increased to 3423 [18].

In Polish law, euthanasia and assisted suicide are prohibited and performing this procedure is punishable by imprisonment from three months to five years (Criminal Code, Art. 150) [19]. The medical Code of Ethics [20] adopted a similar position on the matter of euthanasia and stated that a physician is not allowed to use euthanasia, nor assist a patient in committing suicide (Art. 31).

Attitudes towards euthanasia are associated with the way of understanding the value of life, and what is the maintenance and protection of this value. One of the attitudes, which justifies an objection to euthanasia (lack of acceptance of) stems from the belief that human life is the highest value. In the first half of the 20th century, this attitude was shared by the Alsace philosopher and theologian Albert Schweitzer, creator of the concept of reverence for life, who considered that this idea is a basis of true humanism [21]. He preached not only the need to respect life, but also respect for the will to live and treating it as the highest, absolute value. According to Schweitzer, life as an absolute value is the value in itself, which is good from every point of view, in every relationship, and for every entity. Treating human life as an absolute value is close to the religious perspective, especially Christian philosophy. In accordance with the teachings of the Catholic Church, the special value of human life results mainly from the fact that man was created in the image and likeness of God, therefore, life is a gift from God and no one else can dispose of it [22].

Human dignity and the resulting value of life is understood slightly differently in the rationalist tradition, where the measure of dignity is reason and free will. This is how Immanuel Kant, the 18th century German philosopher, understood the concept of human dignity, claiming that a person can decide about his/her own life and can establish its own moral laws [22]. Some representatives of the humanistic trend in philosophy argue that depriving a person of the right to decide about own death is the violation of individual dignity.

Sociological studies indicate that irrespective of the legal regulations in effect in individual countries, in the last decades, an increase has been observed in the social acceptance of the performance of euthanasia procedures [23, 24]. Analyses performed in Poland by the Centre for Public Opinion Research (CBOS) confirmed the occurrence of such a tendency also in Poland. Nearly 1/3 of Poles (30%) participating in the 1988 study agreed with the opinion that a physician should fulfil the will of a suffering, terminally ill patient who demands the administration of lethal drugs, whereas in 2009, nearly a half of respondents agreed with such an opinion [25]. A study carried out in 2012 showed a decline in the percentage of respondents accepting euthanasia by 5% points (decrease to 43%). Simultaneously, it is noteworthy that in the 2009 study, a nearly two-fold decrease was noted in the percentage of those who were undecided (13.0%), compared to the 1988 study (23.0%). A study by the CBOS showed that during the period 2005–2021, a considerable increase was observed in the percentage of persons who evaluated euthanasia as a morally positive phenomenon [26]. In 2005, such an opinion was expressed by 18% of respondents, almost twice as high a percentage of respondents in 2021–34%). Simultaneously, in 2005, 60% of respondents, and in 2021, 41% evaluated euthanasia as a morally negative phenomenon.

In the context of discussion on euthanasia and the other similar practices, the attitude of young people towards this phenomenon is an extremely interesting problem. The experiences of other countries prove that despite the lack of political will of those in power, the legalization of euthanasia occurs under the influence of public opinion which forces such decisions in referendums [27]. The aim of this study is to determine the degree of acceptance of euthanasia among students from Polish universities across three different fields of study: psychology, medicine, and economic-technical disciplines, and to identify the factors associated with the acceptance of this phenomenon.

A special group in the ongoing debate consists of medical students [28]. However, while research on attitudes towards euthanasia is focused on medical students, psychology students are equally important in this debate. Both medical and psychology studies prepare students for the helping professions, which are aimed at helping others [29]. Despite playing important roles in counselling and health [30], psychologists are often a forgotten group in the debate on euthanasia and other related practices. Current legal regulations and those that may be enacted in the future will impact their actions in helping others. Participating in courses on palliative medicine and medical ethics can provide them with the opportunity to form their own well-considered positions on these issues. Conducting effective courses requires knowledge of their attitudes and the factors influencing these attitudes. Understanding the attitudes of students from disciplines other than medicine and psychology is also very important. Although these studies do not prepare students for work directly related to helping others, the attitudes of students from these fields are significant in referendums deciding on euthanasia. It is also important to provide these students with the opportunity to form their own well-considered positions on these issues.

## Methods

### Participants

The study on attitudes towards euthanasia was conducted with a group of 627 students from three different disciplines at universities in Lublin: medicine, psychology, economic-technical disciplines. The sampling was stratified and purposive. The basis for the stratified division was the selected fields of study at the specified universities. Two criteria were applied, considering the nature of these fields of study (assuming internal homogeneity within each stratum and differences between the strata) and the connection of specific education programs to the subject of the research. The size of each stratum (field of study) was set at approximately 200 students per stratum. Students of the selected disciplines who were present at the university on the day of the survey received a paper questionnaire with a request to complete it. The sample was realized based on the availability of students at the university on the designated survey day and their consent to participate in the research. Ultimately, the study included 280 medical students (14.1% of the total number of students in this field – 1985), 170 psychology students (22.6% of the total number of students in this field – 753), and 177 students of economic-technical disciplines (32.4% of the total number of students in these fields – 546).

### Data collection

The study was conducted using a survey method with an author-constructed questionnaire concerning students’ attitudes towards euthanasia (see Supplementary Material). The questionnaire consisted of 35 items, including six open-ended questions requiring written responses and 29 closed-ended questions with pre-made answer choices. The study examined three components of attitude: knowledge about the phenomenon of euthanasia, evaluation of the phenomenon, and a declaration of behavioral readiness towards euthanasia. The questionnaire also included items concerning the respondents’ demographic characteristics and an assessment of their religious involvement (see Supplementary Material).

### Indicators

Based on the detailed responses, two indicators were developed: (1) the general index of acceptance of euthanasia, and (2) the index of readiness to consent to euthanasia.

**Fig. 1:**
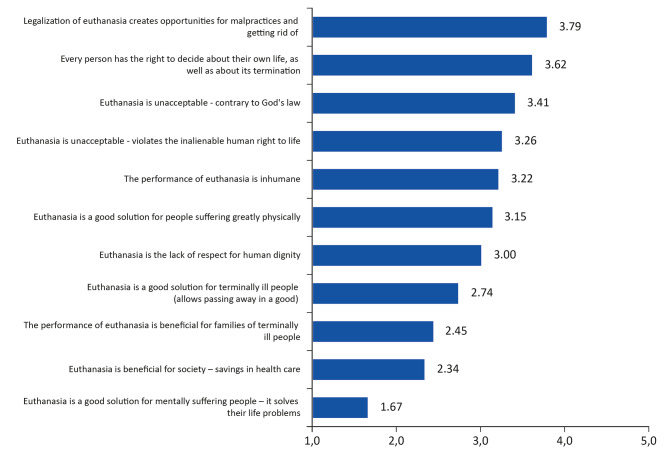
Mean values of evaluations of individual statements concerning euthanasia

Based on 11 statements concerning euthanasia (see Fig. 1), an overall index of acceptance of this phenomenon was created. Statements indicating positive aspects (statements No. 2, 6, 8, 9, 10, 11) obtained point values from 1 to 5, where 1 was the lack of acceptance of the statement, and 5 – full acceptance. Statements indicating negative aspects (statements No. 1, 3, 4, 5, 7) obtained reversed point values from 5 − 1, where 5 was the lack of acceptance for the statement, while 1 – full acceptance. Due to such a differentiation of values ​​​​in scales assessing various types of evaluations (positive and negative), lower values indicated smaller acceptance of euthanasia, and higher – a higher acceptance. For the data prepared in this way, an overall index of acceptance of euthanasia was created, by calculating arithmetic mean based on 11 statements. The values of the new variable remain within 1 and 5, where 1 is the lack of acceptance for euthanasia, and 5 – full acceptance. The reliability of the created index was investigated using Cronbach’s alpha test. Its value − 0.882 - indicates that in accordance with the George and Mallery classification [31], the test items are interconnected, indicating a good internal consistency of the test (α > 0.8). This means that the scale of the acceptance of euthanasia is reliable.

**Fig. 2:**
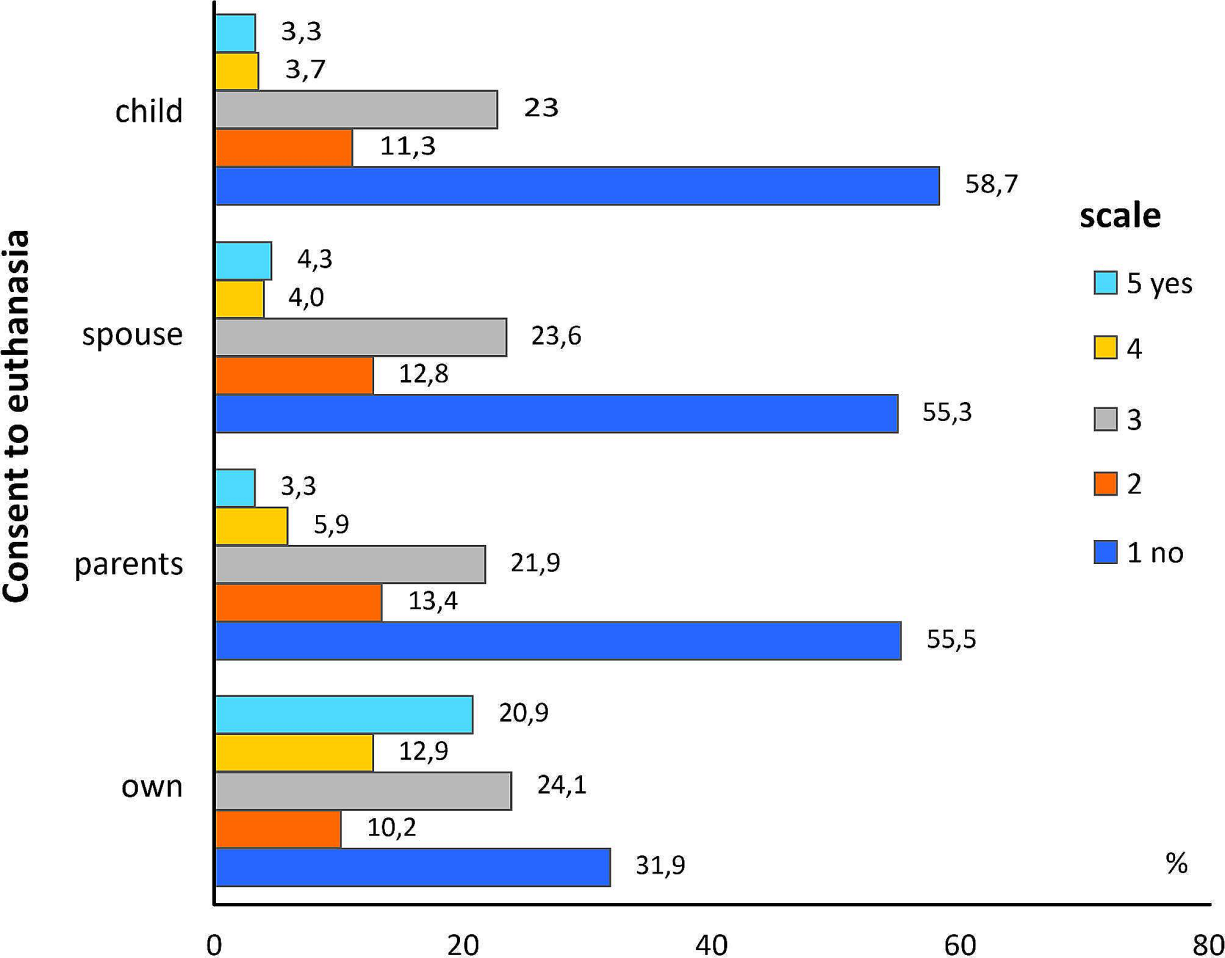
Readiness to express consent for euthanasia – own or family members

Based on the variables concerning readiness to express consent for euthanasia on oneself, parents, spouse, child (see Fig. 2), an overall index of acceptance of this phenomenon was created, calculating the arithmetic mean from the values of the above-mentioned variables. The values of the index remain within 1–5, where 1 is the lack of acceptance for euthanasia, and 5 - full acceptance. The reliability of the index created was investigated using Cronbach’s alpha test. Its value – 0.920 indicated that according to the George and Mallery classification [31], the test items are interconnected, which means a good internal consistency of the test (α > 0.8), and the scale of the index of consent for euthanasia is reliable.

### Data analysis

The analysis of the relationships between variables was conducted using the following methods: Chi^2^, Student’s t-test, Phi test, Cramer’s V test, Kolmogorov-Smirnov test, one-way ANOVA with Dunnett’s post hoc test, linear regression analysis, and categorical regression analysis using optimal scaling (CATREG). The mean acceptance indices were calculated in subgroups distinguished by independent variables. The significance of differences between mean values was examined using Student’s t-test for dichotomous independent variables and one-way ANOVA with Dunnett’s post hoc test for the variable “field of study” (three values). Assumptions for the use of parametric tests were checked prior to analysis. Although the distribution of the acceptance index for euthanasia significantly differs from the normal distribution (K-S=; *p* < 0.001), the skewness is very low (0.08), and there are no extreme values in the distribution. To achieve a distribution closer to the normal distribution, the variable “degree of acceptance of euthanasia” was standardized through logarithmization (logarithm to the base 10). Additionally, the variance of this variable in subgroups distinguished by independent variables allows for the assumption of homogeneity of variances (*p* > 0.05). Considering the verified properties of the data and the sample size (627), the applied tests can be considered reliable. Statistical analyses were performed using SPSS v. 29 software.

## Results

### Demographic characteristics of the respondents

The majority of students in the study were females (69.4%) and urban inhabitants (57.6%) (Table 1). The respondents’ ages varied from 17 to 35, and fell into three age categories slightly differing by numbers: students aged 19 or younger – 38.1% of the total number of respondents, those aged 20–30.0%, and respondents aged 21 or older – 31.9%. The environment of the respondents’ family of origin also varied. The majority of students (62.8%) came from small families with few children (one or two). More than 1/3 of respondents came from large families with several children (37.2%). More than a half of the examined students (58.2%) came from nuclear families (parents and children), while the remainder came from multigenerational families, and had the experience of living together with grandparents at present or in the past.

**Table 1:** Characteristics of examined students

Characteristics of examined students		*N*	%
Gender	Female	435	69.4
	Male	192	30.6
	Total	627	100.0
Place of permanent residence	Urban	361	57.6
	Rural	266	42.4
	Total	627	100.0
Origin from a large family	No (lack of or one sibling)	394	62.8
	Yes (two or more siblings)	233	37.2
	Total	627	100.0
Living together with grandparents in the family	Living at present	179	28.5
	Living in the past	148	23.6
	Has not lived	300	47.9
	Total	627	100.0
Age	Up to 19	239	38.1
	20	188	30.0
	21 and over	200	31.9
	Total	627	100.0
Study discipline	Psychology	170	27.1
	Medicine	280	44.7
	Economic-Technical studies	177	28.2
	Total	627	100.0
Attitude towards religion	Believer - practitioner	396	63.2
	Believer - non-practitioner	153	24.4
	Non-believer	78	12.4
	**Total**	**627**	**100.0**

The respondents were also characterized concerning their attitude towards religion, assuming that this characteristic may be related with attitudes towards euthanasia. The majority of students (63.2%) defined themselves as believers and practitioners. Every fourth respondent (24.5%) reported being a believer non-practitioner, whereas 11.6% of the examined students admitted that they were non-believers. According to these declarations the respondents were divided into two groups: those religiously involved (62.2%), and those not religiously involved (37.8%).

### General assessment of euthanasia

Nearly a half of the examined students (45.9%) evaluated the practice of euthanasia in negative terms, including 25.9% of the total number of respondents as definitely negative, and 21.1% - rather negative (Table 2).

**Table 2:** Overall evaluation of euthanasia according to the respondents’ study disciplines

Evaluation	Study discipline						Total	
	Psychology		Medicine		Economic-Technical			
	*N*	%	*N*	%	*N*	%	*N*	%
Positive	12	7.1	4	1.4	3	1.7	19	3.0
Rather positive	21	12.4	47	16.8	31	17.7	99	15.8
Neither positive nor negative	47	27.6	95	33.9	78	44.6	220	35.3
Rather negative	30	17.6	59	21.1	36	20.6	125	20.0
Negative	60	35.3	75	26.8	27	15.4	162	25.9
**Total**	**170**	**100.0**	**280**	**100.0**	**175**	**100.0**	**625**	**100.0**

Chi^2^ = 35.472; *p* < 0.001

More than twice as small percentage of students evaluated this phenomenon positively (18.8%), including 3% of the total number of respondents – definitely positively, while 15.8% - rather positively. A relatively large number of respondents were unable to make a definite assessment (33.9%) claiming that euthanasia cannot be ascribed either a positive nor a negative evaluation. Evaluations of the phenomenon of euthanasia significantly differed in sub-groups distinguished according to the study discipline, and this concerned mainly negative evaluations. A similar percentage of students of psychology (19.5%), medicine (18.2%) and technical disciplines (19.4), evaluated euthanasia positively or rather positively. The greatest differences were observed among persons evaluating euthanasia definitely negatively. Such evaluations of this phenomenon were reported by 35.3% students of psychology, a considerably smaller percentage of students of medicine (26,8%), and the smallest percentage of those studying technical disciplines (15.4%). Considering negative and rather negative evaluations, also in the group of students of technical disciplines, the percentage of these evaluations was the lowest (36%), while the highest among students of psychology (52.9%). The lowest percentage of those indecisive was observed among students of psychology (27.6%), indecision was declared by a slightly higher percentage of students of medicine, and the highest percentage of students of technical disciplines (44.6%). The observed differences were statistically significant (*p* < 0.001).

The students in the study were presented with 11 opinions formulated by opponents and supporters of euthanasia, asking them to evaluate to what extent they agree with their content. They were asked to evaluate these opinions according to a 5-point scale, where 1 - lack of acceptance of the statement, and 5 - full acceptance. Two statements representing contradictory attitudes concerning euthanasia obtained the greatest acceptance. Opponents of euthanasia, while justifying their attitudes, indicated that its legalization creates opportunities for malpractices, and getting rid of inconvenient and useless people. This statement obtained the highest mean evaluation (3.79) (Fig. 1).

The statement by supporters of euthanasia that every person has the right to decide about their own life, as well as about its termination, received slightly less recognition (M = 3.62). The three subsequent statements obtained a high mean value concerning the lack of acceptance of such a type of practice: ‘euthanasia is unacceptable because it is contrary to God’s law’ (M = 3.41), ‘euthanasia is unacceptable because it violates the inalienable human right to life’ (M = 3.26), and ‘the performance of euthanasia is inhumane’ (M = 3.22).

One more statement indicating a positive attitude towards euthanasia: ‘euthanasia is a good solution for people suffering terribly physically - it relieves them of suffering’, and the statement opposing euthanasia, ‘euthanasia is the lack of respect for human dignity’, obtained a relatively high degree of acceptance (M = 3.15 and M = 3.0). The remaining statements obtained considerably lower degrees of acceptance (below the value 3 – median value on the scale from 1 to 5), and all of them concern negative evaluation of this phenomenon. The examined students to the lowest degree accepted the opinion that ‘euthanasia is a good solution for mentally suffering people – it solves their life problems’ - M = 1.67, where 1 is the lack of acceptance.

### Acceptance of euthanasia and its predictors

A comparison of the general index of acceptance of euthanasia (for the method of calculation, see Indicators) among groups of students categorized by field of study showed that the level of acceptance of this phenomenon in at least one group significantly differs from the levels of acceptance in the other groups (one-way analysis of variance: F = 4.57; *p* < 0.05) (Table 3). The use of Dunnett’s *post hoc* test showed which pairs of values significantly differed. Analysis of the test indicated that the level of acceptance of euthanasia in the groups of students of psychology and medicine was very similar (no significant differences). Considerable differences in this respect occurred between students of psychology and those studying economic-technical disciplines. The level of acceptance of euthanasia in the group of students of psychology was significantly lower, compared to students of economic-technical disciplines (*p* < 0.05). Students of medicine, to a lower degree accepted euthanasia (M = 2.63), compared to students of economic-technical disciplines (M = 2.82) (the difference was insignificant, although close to significant (*p* = 0.058).

**Table 3:** Overall index of acceptance of euthanasia according to the respondents’ study disciplines

Study discipline	Descriptive statistics			One-way ANOVA		Dunett’s post hoc test			
	*N*	M	DS	F	*p*	Categories compared	difference M	SD	*p*
1) Psychology	170	2.55	0.93	4.57	0.011	1–2	-0.08	0.09	0.723
2) Medicine	280	2.63	0.89			1–3	-0.28	0.09	0.012
3) Economic - Technical	177	2.82	0.85			2–3	-0.19	0.08	0.058
**Total**	**627**								

The relationships observed in bivariate analyses do not include the simultaneous effect of other variables, therefore, in order to determine the model of the conditioning of the level of acceptance of euthanasia among students of the selected disciplines, multivariate linear regression analysis was applied. In the theoretical model, the dependent variable is the quantitative variable – level of acceptance of euthanasia. Into the model were included, by the method of introduction, eight independent variables, including one quantitative – respondent’s age (number of years with an accuracy of one year) and seven dichotomized variables: gender (one female; two males), place of permanent residence (one rural; two urban), origin from a large family (1 - No; 2 - Yes), living with generation of grandparents (1 - No; 2 - Yes), religious involvement (1 - not involved; 2 - involved), student of psychology (1 - No; 2 - Yes), student of medicine (1 - No; 2 - Yes). Correlation matrix of independent variables demonstrated that some pair of variables were significantly correlated, but the correlations were very weak or weak (values of correlation coefficient Cramer’s Phi/V (for the variable of age) from 0.017 to 0.360). A relatively high correlation coefficient was observed between the variables studying psychology and studying medicine (-0.548); however, this value was much smaller than the value 0.7, at which it is recommended to remove one pair of variables from the model [20]. By adopting this principle, both variables were left in the model. The variable of being a student of an economic-technical discipline was not introduced into the model, treating this category as the reference for the remainder.

**Table 4:** Determinants of the degree of acceptance of euthanasia (linear regression)

Predictors (coding)	ß	SE	Stand. ß	t	*p*	95% CI for B		VIF
						lower	upper	
(Constant)	3.837	0.303		12.654	< 0.001	3.241	4.432	
Repondent’s age	-0.084	0.067	-0.046	-1.253	0.211	-0.216	0.048	1.182
Gender (1 - female, 2 -male)	0.079	0.068	0.041	1.165	0.244	-0.054	0.212	1.073
Place of permanent residence (1 - urban, 2 - rural)	0.02	0.066	0.011	0.301	0.764	-0.110	0.15	1.191
Origin from large family (1 - No, 2 - Yes)	-0.054	0.065	-0.029	-0.826	0.409	-0.181	0.074	1.091
Living with grandparents (1 - No, 2 - Yes)	0.026	0.062	0.014	0.415	0.679	-0.096	0.148	1.073
Religious involvement (1 - No, 2 - Yes)	-0.998	0.065	-0.537	-15.409	< 0.001	-1.125	-0.871	1.077
Student of psychology (1 - No, 2 - Yes)	-0.251	0.088	-0.125	-2.858	0.004	-0.423	-0.078	1.697
Student of medicine (1 - No, 2 - Yes)	-0.13	0.078	-0.072	-1.664	0.097	-0.283	0.023	1.665

ANOVA for regression: F = 34.555; *p* < 0.001; Explained variability of the dependent variable, corrected R^2^ = 0.302

Linear regression analysis emerged as an empirical model containing to variables significantly related with the level of acceptance of euthanasia: religious involvement and studying psychology (Table 4). This model is statistically significant (F = 34.555; *p* < 0.001), and its predictors explain 30% of the variability of the level of acceptance of the phenomenon of euthanasia (corrected R^2^ = 0.302). Both predictors of the model, both being a religious person and being a student of psychology, exerted an effect on the smaller acceptance of euthanasia. Comparison of standardized coefficients β for both variables indicate that religiosity is considerably more strongly related with the degree of acceptance of euthanasia (β=-0.537) than studying psychology (β=-0.125).

### Predictors of readiness for expression of consent for euthanasia

The indicator of acceptance of euthanasia in terms of specified behaviours or readiness for such behaviours, is readiness to express consent for euthanasia on oneself and close family members. The examined students evaluated such readiness according to the 5-point scale, where 1 was lack of consent for euthanasia, and 5 full consent. The evaluations concerned euthanasia on oneself, parents, spouse, and own child. Analyses of the replies obtained showed that 1/5 of respondents would express their consent for the performance of euthanasia on oneself (value 5 according to 5-point scale), and 12.9% of respondents would rather not express consent for euthanasia (value 4) (Fig. 2). A considerably smaller number of respondents adopted such an attitude (values 5 and 4) on their closest relatives: parents − 9.2%, spouse − 8.3%, and own child − 7.0%. Simultaneously, more than a half of respondents declared that they would not express their consent for euthanasia on their close relatives: child − 58.7%, spouse − 55.8, parents − 55.3%. As many as 3.9% of the total number of respondents definitely excluded the performance of euthanasia on oneself.

In order to determine predictors affecting readiness for expression of consent for euthanasia, the CATREG optimal scaling analysis was used as an alternative for linear regression, because the distribution of the independent variable differs from normal distribution. In the theoretical model, the dependent variable is a quantitative variable – index of readiness to express consent for euthanasia. Eight independent variables were included into the model: respondents’ age, gender, place of permanent residence, origin from a large family, living with the generation of grandparents, religious involvement, studying psychology, and studying medicine. These are the same variables as those introduced into the model of conditioning of the level of acceptance of euthanasia (linear regression).

The CATREG optimal scaling analysis emerged as an empirical model containing four variables significantly related with readiness to express consent for euthanasia: religious involvement (*p* < 0.001), living with the generation of grandparents (*p* < 0.001), origin from a large family (*p* < 0.05), and studying psychology (*p* < 0.05) (Table 5).

This model is statistically significant (F = 17.368; *p* < 0.001), and its predictors explain 19.1% of variability of readiness to express consent for euthanasia (corrected R^2^ = 0.191). Readiness to express consent for euthanasia was negatively related with the respondents’ religious involvement, studying psychology, and origin from a large family (Table 5).

**Table 5:** Determinants of the index of readiness for expression of consent for euthanasia on oneself or close relatives (categorical regression analysis using the optimal scaling method (CATREG))

Predictor (coding)					Significance of the model	
	β	F	p	corrected R^2^	F	p
Gender (1 fermale, 2 male)	-0.034	1.495	0.222	0.191	17.368	< 0.001
Respondent’s age (1 younger, 2 older)	0.022	0.327	0.568			
Students of psychology (1- No, 2 - Yes)	-0.087	3.892	0.049			
Students of medicine (1 - No, 2 - Yes)	-0.038	1.214	0.271			
Religious involvement (scale from 1–3)	-0.419	117.45	< 0.001			
Living with grandparents (1 - No, 2 - Yes)	0.100	8.562	< 0.001			
Origin from a large family (1 - No, 2 - Yes)	-0.077	4.652	0.031			
Place of permanent residence (1 urban, 2 rural)	-0.002	0.003	0.958			

Students of psychology who were religiously involved, and those coming from large families, to a significantly lower degree were ready to express their consent for euthanasia. Respondents who had the experience of living with the generation of grandparents showed a greater tendency to perform euthanasia (β = 0.100; *p* < 0.001). Religiosity was the strongest predictor of consent for euthanasia (β=-0.419).

## Discussion

In some countries worldwide, euthanasia or assisted suicide are legally allowed. In Poland, practices in this area are prohibited. Despite this, studies of attitudes towards euthanasia demonstrated that in recent decades an increase has been observed in Polish society in the acceptance of euthanasia as a way to shorten the suffering of terminally ill people [26]. In 2021, 1/3 of Poles evaluated euthanasia in positive terms. The results of the presented study among Polish students show that approximately 20% declared readiness to express consent for euthanasia on oneself, and less than 1/3 definitely rejected such a possibility. More than a half of respondents were definitely against euthanasia on their closest family members. Similar results were obtained by Szadowska-Szlachetka et al., who conducted a study among students of nursing [32]. The researchers confirmed that nearly a half of the examined students were definitely against legalization of euthanasia in Poland, 40.8% declared that they would not agree to the performance of euthanasia on a terminally ill loved one, whereas with respect to oneself in the situation of incurable illness, every third student would not perform euthanasia, whereas 22.5% of student would take such a step.

Slightly different results were obtained by W. Leppert et al. who investigated Polish 4th year medical students, and found that in the case of an incurable illness, euthanasia oneself or the close ones, 17.1% of the respondents would chose euthanasia, and 20.9% - assisted suicide [33]. The level of acceptance of euthanasia among students of medicine is considerably higher in New Zealand than in Netherlands [27]. A study conducted in 2018 showed that 56% of medical students support the legalization of euthanasia, while 22% are against it.

In New Zealand, support for legalization of euthanasia decreases among students in the later years of a study. Legalization of euthanasia was supported by 64.8% of the 2nd year students, whereas among 5th year students, the percentage of supporters of euthanasia decreased down to 39.1% (difference by 25.7% point) [27]. In India, a study carried out among students of medicine and nursing also showed a high level of support of euthanasia (61%), although, at the same time, they admitted ethical dilemmas concerning euthanasia and its legalization [34]. Similar results were obtained among Serbian medical students and showed that more than 50% of 2nd year students, and 60% of 5th year students supported euthanasia; simultaneously, 5th year students 2.5 times more often expressed the conviction that euthanasia should be clearly legally regulated [35]. It could be presumed that acquisition of medical knowledge by students increases the level of reflection on euthanasia, and may also decrease the level of its acceptance.

A study carried out among Belgian students also indicated differences in attitudes towards euthanasia according to study discipline. Acceptance of euthanasia was the lowest among students of medicine (31% fully accepted), compared to an identical attitude of students of philosophy (56% accepted euthanasia), and students of law (47%) [36].

Based on the analysis of the results of the presented study, it was found that the level of acceptance of euthanasia by students varied according to the study discipline. Students of economic-technical disciplines accepted euthanasia to a higher degree, whereas students of psychology to the lowest degree. Multivariate analysis confirmed a significant relationship between acceptance of euthanasia and studying psychology, and showed that study of this discipline was significantly related with a lower level of acceptance of euthanasia.

A study conducted in 62 countries worldwide within the international project The World Values Survey (WVS), demonstrated that in the majority of the examined countries religion exerts a significant effect on attitudes towards euthanasia [37]. In many other studies, religious involvement was a significant predictor of acceptance of euthanasia [27, 38–40]. This regularity was also confirmed by the presented study which showed that religiously involved students, to a significantly smaller extent, were ready to express their consent for euthanasia on oneself or closest relatives, and accepted euthanasia to a lower degree than those not religiously involved. In addition, religious involvement was the predictor most strongly related with various dimensions of attitudes towards euthanasia (β =-0.419 -0.537).

The current study of students also demonstrated that coming from a large family and the experience of living with the generation of grandparents was a significant predictor of readiness to express consent for euthanasia on oneself or closest relatives. Respondents coming from small families (one sibling or none) showed a greater readiness in this respect, as well as persons who live or had lived with people at old age - grandmother, grandfather.

A more positive attitude towards euthanasia among individuals who have experience living with grandparents may result from observing elderly people suffering from incurable severe illnesses. The lack of adequate care for such individuals causes great suffering and helplessness among their loved ones. This situation may influence the liberalization of views on euthanasia. This assumption is supported by studies conducted in Poland, which indicate that the medical staff’s contact with terminally ill, suffering patients may contribute to their support for the legalization of euthanasia [41].

## Conclusions

Summing-up, it can be concluded that despite many studies concerning attitudes towards euthanasia, its forms, legalization, and the level of acceptance, it still remains a great challenge of ethical-moral, medical, and legal character. Simultaneously, there is a great diversity of attitudes towards euthanasia in societies with different political, religious and cultural systems. The great importance of Christianity in the lives of most Poles, especially Catholicism, and legal ban on euthanasia explain the lower acceptance of any form of euthanasia, compared to other West European countries. However, taking into account the ongoing process of the secularization of Polish youth [42], it could be expected that the level of acceptance of euthanasia will increase in this social group, and in the whole of society. The variation in the level of acceptance of euthanasia among student groups from different fields of study suggests that knowledge about the psycho-somatic functioning of humans plays a significant role in shaping attitudes towards such practices. The lower level of acceptance of euthanasia among psychology and medical students compared to students of economic-technical fields indicates that the curricula of these studies present alternative solutions to the problems of terminally ill patients other than euthanasia. Understanding the latest advancements in palliative medicine is undoubtedly an important element of a rational approach to addressing issues related to old age and suffering.

The presented study shows characteristics of the family environment of Polish students (origin from a large family, living with the generation of grandparents) as predictors of attitudes towards euthanasia, which has not been confirmed in studies by other researchers. The explanation of these relationships requires further in-depth research taking into consideration not only social, but also psychological variables.

A limitation of these studies is their representativeness. The research was conducted in one city among students from three fields of study. It would be advisable to conduct research in selected academic centers across Poland, including a larger number of study disciplines.

### Electronic supplementary material

Below is the link to the electronic supplementary material.

Supplementary Material 1

Supplementary Material 2

## Data Availability

The authors confirm that the data supporting the findings of this study are available within the article and its supplementary materials.

## Abbreviations

CATREG: Categorical Regression analysis using the Optimal Scaling method
