# Supplementary material for: Acceptance of euthanasia by students of selected study disciplines at universities in Lublin, Poland
Source: BMC Med Ethics. 2024 Jul 26;25:83. doi: 10.1186/s12910-024-01071-7 (PMC11282810; doi:10.1186/s12910-024-01071-7)
Supplement: Supplementary file 1 — Supplementary Material 1 [file 12910_2024_1071_MOESM1_ESM.docx]

Department of Social Research Methods

Institute of Sociology

Maria Curie-Skłodowska University

Anonymous survey

On the topic:

Attitudes of Students Towards Euthanasia

We invite you to participate in a survey of students from Lublin universities. The aim of the research is to understand the attitudes of young people towards euthanasia. Completing the survey involves entering responses in the provided spaces or selecting the chosen response from the sets provided in the survey. The survey is anonymous, and its results will be used for preparing scientific papers.

1. Which of the terms provided in the table correctly define the concept of euthanasia? Place an "X" in the column indicating the chosen answer.

| Terms of euthanasia | Yes | No | I don't know |
| --- | --- | --- | --- |
| 1. Euthanasia is a good, peaceful death, without suffering, caused by old age. |  |  |  |
| 1. Euthanasia is the shortening of a patient's life by a doctor in a very serious or hopeless condition by disconnecting from life-support equipment (artificial nutrition, breathing, and circulation). |  |  |  |
| 1. Euthanasia is the shortening of a patient's life by medical staff in a very serious or hopeless condition by discontinuing treatment. |  |  |  |
| 1. Euthanasia is the unintentional (accidental) causing of death to a severely ill person due to a medical error or overdose of pain-relieving drugs. |  |  |  |
| 1. Euthanasia is the administration of pain-relieving drugs to a person in a very serious or hopeless condition to provide a peaceful death free from suffering. |  |  |  |
| 1. Euthanasia is the killing by medical staff of a patient suffering from an incurable disease, very much in pain, by administering a large dose of pain-relieving medication. |  |  |  |
| 1. Euthanasia is the administration by family members of a large dose of pain-relieving medication to a person suffering from an incurable disease, in severe pain, in order to shorten their suffering. |  |  |  |
| 1. Euthanasia is suicide committed by a person suffering from an incurable disease causing unbearable suffering. |  |  |  |
| 1. Euthanasia is performed at the request of assistance in suicide to people very old, physically and mentally disabled, of no use to anyone, and not needing anyone. |  |  |  |
| 1. Another definition that you find more accurate:………………………………………… |  |  |  |

1. In Poland, is euthanasia legally permitted? Please check all true answers.
2. Yes, when requested by the sick person
3. Yes, when requested by the family of the sick person
4. Yes, when the decision is made by a specially appointed council
5. Yes, when the decision is made by the court
6. Yes, but after additional circumstances arise – what …………………………………
7. No, euthanasia is prohibited
8. I don't know
9. In your opinion, are euthanasia procedures carried out in Poland?
10. Definitely yes
11. Probably yes
12. Hard to say
13. Probably not
14. Definitely not
15. If euthanasia procedures are performed in Poland, how do you estimate the scale of this phenomenon?

Please enter the approximate number of euthanasia procedures performed per year.

A. Number of legal euthanasia procedures …………………………

B. Number of illegal euthanasia procedures …………………………

1. Is euthanasia legally permitted in other European countries?
2. Yes - in all countries (skip to question 7)
3. Yes - in most countries
4. Yes - but only in some
5. No - it is prohibited in all European countries (skip to question 7)
6. I don't know (skip to question 7)
7. In which European countries is euthanasia legally permitted?

…………………………………………………………………………………………………

1. In which countries in the world are the most euthanasia procedures performed? Please enter three countries where the most such procedures are performed.
2. I don't know
3. ……………………………………
4. ……………………………………
5. ……………………………………
6. If euthanasia is prohibited in a certain country, do you think this type of practice should be legalized?
7. Definitely yes
8. Probably yes
9. Hard to say
10. Probably not
11. Definitely not
12. If you believe that euthanasia should be legalized in countries where there are no such regulations, who do you think should decide on euthanasia? You can choose more than one answer.
13. The patient themselves
14. The doctor
15. The patient's closest family
16. The court
17. Other individuals/institutions (who?)……………………………………………..
18. Please rate the extent to which you agree with the statements below regarding euthanasia. Please rate each statement on a 5-point scale, where 1 indicates strongly disagree (with the statement), and 5 indicates strongly agree (with the statement). Please circle the chosen number on the scale.

A. Every person has the full right to decide about their own life, including its end.

| 1  Strongly disagree | 2 | 3 | 4 | 5  Strongly agree |
| --- | --- | --- | --- | --- |

1. Eutanazja jest dobrym rozwiązaniem dla osób nieuleczalnie chorych, ponieważ pozwala odejść z tego świata w stosunkowo dobrej kondycji

| 1  Strongly disagree | 2 | 3 | 4 | 5  Strongly agree |
| --- | --- | --- | --- | --- |

1. Euthanasia is unacceptable because it violates the inherent human right to life

| 1  Strongly disagree | 2 | 3 | 4 | 5  Strongly agree |
| --- | --- | --- | --- | --- |

1. Euthanasia is a good solution for people who suffer greatly physically because it shortens their suffering

| 1  Strongly disagree | 2 | 3 | 4 | 5  Strongly agree |
| --- | --- | --- | --- | --- |

1. Euthanasia is unacceptable because it contradicts divine law.

| 1  Strongly disagree | 2 | 3 | 4 | 5  Strongly agree |
| --- | --- | --- | --- | --- |

1. Euthanasia is a good solution for people who suffer greatly mentally because it resolves their life problems

| 1  Strongly disagree | 2 | 3 | 4 | 5  Strongly agree |
| --- | --- | --- | --- | --- |

1. Euthanasia is inhumane

| 1  Strongly disagree | 2 | 3 | 4 | 5  Strongly agree |
| --- | --- | --- | --- | --- |

1. The use of euthanasia is beneficial for families of terminally ill individuals

| 1  Strongly disagree | 2 | 3 | 4 | 5  Strongly agree |
| --- | --- | --- | --- | --- |

1. Euthanasia is a lack of respect for human dignity

| 1  Strongly disagree | 2 | 3 | 4 | 5  Strongly agree |
| --- | --- | --- | --- | --- |

1. Legalizing euthanasia creates opportunities for abuse and getting rid of inconvenient or useless individuals

| 1  Strongly disagree | 2 | 3 | 4 | 5  Strongly agree |
| --- | --- | --- | --- | --- |

1. Euthanasia is beneficial for society because it leads to savings in the healthcare and pension system

| 1  Strongly disagree | 2 | 3 | 4 | 5  Strongly agree |
| --- | --- | --- | --- | --- |

1. How do you generally assess the use of euthanasia?
2. Strongly positively
3. Rather positively
4. Neither positively nor negatively
5. Rather negatively
6. Strongly negatively
7. If you assess euthanasia positively, what are the benefits associated with its use? …………………………………………………………………………………………………………………………………………………………………………………………………………
8. If you assess euthanasia negatively, what are the risks associated with its use? …………………………………………………………………………………………………………………………………………………………………………………………………………
9. In your opinion, should doctors fulfill the wishes of terminally ill patients who demand to be given drugs that cause death?
10. Strongly yes
11. Rather yes
12. Hard to say
13. Rather no
14. Strongly no
15. In your opinion, should doctors fulfill the wishes of the family of a person in a coma, with no possibility of waking up, to disconnect them from life support equipment?
16. Strongly yes
17. Rather yes
18. Hard to say
19. Rather no
20. Strongly no
21. Which of the listed emotions do you feel towards people who have undergone euthanasia? Please place an "X" in the column indicating the chosen answer.

|  | Yes | Rather yes | Hard to say | Rather no | No |
| --- | --- | --- | --- | --- | --- |
| Respect |  |  |  |  |  |
| Compassion |  |  |  |  |  |
| Sympathy |  |  |  |  |  |
| Admiration |  |  |  |  |  |
| Contempt |  |  |  |  |  |
| Anger |  |  |  |  |  |

1. Which of the listed emotions do you feel towards individuals who perform euthanasia? Please place an "X" in the column indicating the chosen answer.

|  | Yes | Rather yes | Hard to say | Rather no | No |
| --- | --- | --- | --- | --- | --- |
| Respect |  |  |  |  |  |
| Compassion |  |  |  |  |  |
| Sympathy |  |  |  |  |  |
| Admiration |  |  |  |  |  |
| Contempt |  |  |  |  |  |
| Anger |  |  |  |  |  |

1. Would you consent to your own euthanasia or the euthanasia of members of your family?

|  | Yes | Rather yes | Hard to say | Rather no | No |
| --- | --- | --- | --- | --- | --- |
| Self-euthanasia |  |  |  |  |  |
| Euthanasia of parents |  |  |  |  |  |
| Euthanasia of spouse |  |  |  |  |  |
| Euthanasia of one's own child |  |  |  |  |  |

1. If you were willing to consent to your own euthanasia, under what circumstances would you make such a decision?
2. Terminal illness
3. Complete paralysis of the body
4. In the case of a disease causing persistent pain and suffering
5. In the case of artificial life support
6. Other (what?)……………………………………….
7. If you were willing to consent to the euthanasia of a close person to you, under what circumstances would you make such a decision?

………………………………………………………………………………………………………………………………………………………………………………………………………………………………

1. In some countries, such as Belgium, there is the possibility of signing a so-called "Living Will." This is a declaration in which one states that in the event of permanent loss of consciousness, they do not wish for life-sustaining measures to be taken. Do you think such a solution should be adopted in Polish law?
2. Strongly yes
3. Rather yes
4. Hard to say
5. Rather no
6. Strongly no
7. Would you decide to sign such a declaration (Living Will)?
8. Strongly yes
9. Rather yes
10. Hard to say
11. Rather no
12. Strongly no
13. In countries where euthanasia is legal and practiced, some elderly people, fearing for their lives, permanently move to countries where euthanasia is prohibited. Do you think these fears are justified?
14. Strongly yes
15. Rather yes
16. Hard to say
17. Rather no
18. Strongly no
19. If you lived in a country where euthanasia is legal and practiced, would you, being elderly, stay in that country or would you move to a country where euthanasia is prohibited?
20. I will stay
21. Rather I will stay
22. I don't know
23. Rather I will leave
24. I will leave
25. Would you decide to sign such a declaration (Living Will)?
26. Strongly yes
27. Rather yes
28. Hard to say
29. Rather no
30. Strongly no
31. Several American doctors have been assisting terminally ill patients and those suffering from unbearable pain in shortening their lives for several years. How do you assess the actions of these doctors?
32. Strongly positively
33. Rather positively
34. Hard to say
35. Rather negatively
36. Strongly negatively
37. In one of the hospitals in Western Europe, there was a situation where a completely paralyzed young athlete, who had no chance of even the slightest improvement in health, decided to undergo euthanasia. This was made possible in the hospital by administering appropriate drugs. In your opinion, does the action of the medical staff - fulfilling the patient's will - deserve more to be called an act of mercy or rather murder?
38. It was an act of mercy
39. It was an act of murder
40. Hard to say
41. I assess it differently (how?)……………………………………………..

**Demographic data**

1. Age………………………………………
2. Gender
   1. Female
   2. Male
   3. Another answer
3. Field of study………………………………….
4. Year of study………………………………………..
5. Place of permanent residence (origin):………………..
   1. City
   2. Village
6. How would you rate your level of religious involvement?
   1. Religious - practicing
   2. Religious - non-practicing
   3. Non-religious
7. Did your grandparents live in your family home?
   1. Yes, and they still do
   2. Yes, but they no longer do
   3. No
8. Do you have siblings?
   1. No
   2. Yes (enter the number of siblings)…………..
